# The Potential of Graphene as an Adsorbent for Five Pesticides from Different Classes in Rape Oil Samples Using Dispersive Solid-Phase Extraction

**DOI:** 10.1155/2018/3587860

**Published:** 2018-04-01

**Authors:** Katarzyna Madej, Katarzyna Janiga, Wojciech Piekoszewski

**Affiliations:** ^1^Department of Analytical Chemistry, Faculty of Chemistry, Jagiellonian University, Krakow 30-387, Poland; ^2^Department of Food Science and Technology, School of Biomedicine, Far Eastern Federal University, Vladivostok, Russia

## Abstract

Isolation conditions for five pesticides (metazachlor, tebuconazole, *λ*-cyhalothrin, chlorpyrifos, and deltamethrin) from rape oil samples were examined using the dispersive solid-phase graphene extraction technique. To determine the optimal extraction conditions, a number of experimental factors (amount of graphene, amount of salt, type and volume of the desorbing solvent, desorption time with and without sonication energy, and temperature during desorption) were studied. The compounds of interest were separated and detected by an HPLC-UV employing a Kinetex XB-C18 column and a mobile phase consisting of acetonitrile and water flowing in a gradient mode. The optimized extraction conditions were: the amount of graphene 15 mg, desorbing solvent (acetonitrile) 5 mL, time desorption 10 min at 40°C, and amount of NaCl 1 g. The detection limit for metazachlor, tebuconazole, *λ*-cyhalothrin, and chlorpyrifos was 62.5 ng·g^−1^, and for deltamethrin, it was 500 ng·g^−1^. The obtained results lead to the conclusion that graphene may be successfully used for the isolation of the five pesticides from rape oil. However, their determination at low concentration levels, as they occur in real oil samples, requires the employment of appropriately highly sensitive analytical methods, as well as a more suitable graphene form (e.g., magnetically modified graphene).

## 1. Introduction

Due to the potential toxicity, bioaccumulating properties, and wide use of pesticides for supporting and intensification of crops almost all over the world, these substances belong to the most often controlled ones in the environment and in food. Every year, many publications devoted to the development of new methods for pesticide determination in various food matrices appear. However, pesticide residue analysis in high-fat samples is still a challenging analytical task. Edible oil samples belong to such analytically difficult matrices. Due to complex matrices, as well as low concentrations of pesticide residues, frequently originated from various classes, these samples are analyzed using such advanced coupled techniques as LC-MS/MS [1, 2], LC-TOF-MS [3], UHPLC-MS/MS [4, 5], GC-MS/MS [2, 6], and GC-MS [7]. The employment of GC-ECD [8, 9], HPLC/UV [10], or HPLC/DAD [11] in this analytical area has also been reported. Generally, in all cases of oil matrix analysis, an appropriate sample preparation procedure, usually consisting of two or more stages, was required. In most cases, QuEChERS or modified QuEChERS-based procedures as the cleanup step were involved [1–6, 8]. In some cases, these were preceded by the freezing step for oil precipitation [2, 3, 8, 10]. The separate review article concerning recent developments and trends in the QuEChERS sample preparation approach, including the analysis of pesticides in various fatty food samples, was also presented [12].

Graphene is a carbon nanomaterial which has been widely used (especially in modified forms) in determination of organic compounds including pesticides, usually in relatively simple matrices. These include environmental waters [13–16], fruit juices [17], fruits [18], vegetables [18–20], and teas [21, 22]. The potential unique adsorption properties of graphene resulted mainly from its large surface area and strong *π*-*π* interactions between its large delocalized electron system and the aromatic rings of the target molecules [23].

**Figure 1:**
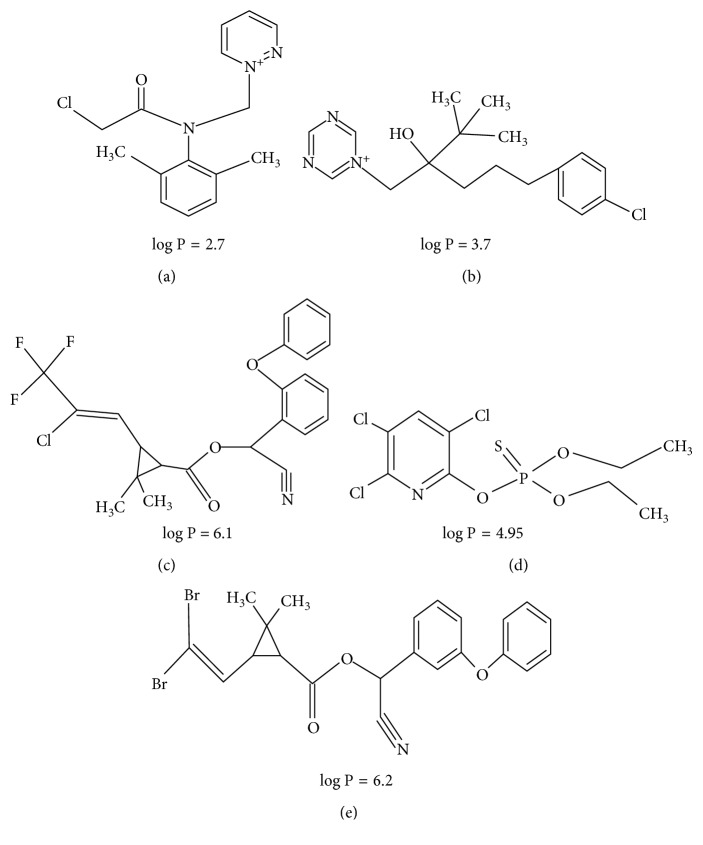
Chemical structures of the studied five pesticides with their partition coefficients (log  *P*). Metazachlor (log  *P*=2.7) (a); tebuconazole (log  *P*=3.7) (b); *λ*-cyhalothrin (log  *P*=6.1) (c); chlorpyrifos (log  *P*=4.95) (d); deltamethrin (log  *P*=6.2) (e).

In this work, the main focus was to evaluate the adsorption potential of nonmodified graphene for the isolation of selected pesticides from various groups (chloroacetamide herbicides, triazole fungicides, pyrethroids, and organophosphorus pesticides) present in rape oil samples, which are commonly used for supporting of rape crops. To our best knowledge, this is the first time that graphene has been tried as the adsorbent for isolation of pesticides from such a difficult matrix as edible oil. The chemical structures of the studied components are presented in Figure 1.

Dispersive solid-phase extraction (d-SPE) combined with graphene was selected as the sample treatment method, and the HPLC-UV technique was used for the separation and detection of the compounds of interest in the resulting oil extracts.

## 2. Materials and Methods

### 2.1. Preparation of Standard Samples and Graphene Suspension

The stock of methanolic solutions of metazachlor, tebuconazole, *λ*-cyhalothrin, chlorpyrifos, and deltamethrin (1 mg·mL^−1^) and the internal standard chlorfenvinphos (1 mg·mL^−1^) were stored in a refrigerator at 4°C. The standard solutions were prepared by appropriate dilution of the stock solutions with methanol. The graphene suspension (3 mg·mL^−1^) was prepared by weighing an appropriate amount of solid graphene and dispersing it in a suitable amount of deionized water using sonication for 6 h. The oil samples were prepared by weighing 1 gram of a commercial, ratified rape oil and then spiking it with appropriate amounts of standard solutions of the five or six pesticides and then left for equilibration at room temperature for one hour.

### 2.2. Apparatus

For oil sample preparation, the following laboratory equipment was used: technical balance (Radwag WPX 250, Poland), vortex TK 35 (Techno-Kartel, Germany), centrifuge MPW-260R (DHN, Poland), thermoblock TB-941U (Poland), and sonic bath Vibra Cell (Sonics & Materials Inc., USA). Chromatographic analyses were performed using the chromatographic system (Merck-Hitachi LaChrom) consisting of an L-7100 pump and an L-7455 UV spectrophotometric detector (Darmastadt, Germany) equipped with a Kinetex XB-C18 column (150 × 4.6 mm, 5 *µ*m) which was supplied by Phenomenex (USA). The column temperature was 25°C. A mixture of water (A) and acetonitrile (B) was used as the mobile phase. The following optimal gradient conditions of the mobile phase flow were applied: 0 min (70% A and 30% B), 20 min (24% A and 76% B), 25 min (0% A and 100% B), and 35 min (0% A and 100% B). The flow rate was 1 mL·min^−1^. The pesticides were detected at *λ* = 220 nm. The injection volume of standard solutions as well as oil sample extracts was 40 *µ*L.

### 2.3. General d-SPE (Graphene) Procedure

For initial isolation of the five pesticides, 4 mL of acetonitrile was added with 1 g pesticide-spiked oil, vortexed (1 min), and left to freeze for minimum 2 h at −32°C in a horizontal position. An acetonitrile supernatant was decanted into a clean centrifuge test tube (7 mL) from above the frozen fat layer. The supernatant was evaporated to almost dryness, and then, an appropriate amount of aqueous graphene suspension and solid sodium chloride (for salting out pesticides) was added. The whole content of the test tube was vortexed (1 min) and then centrifuged (10 min, 14,000 rpm). Using a Pasteur pipette, the upper layer was discarded, and 4 mL of desorbing solvent was added to the bottom layer of graphene with the adsorbed pesticides and vortexed for 1 min without previous sample heating or after increasing the sample temperature in a thermoblock. Subsequently, the sample was subjected to sonication for an appropriate time at an appropriate temperature. The content was centrifuged (10 min, 14,000 rpm), and the acetonitrile supernatant with desorbed compounds was successfully transferred into an Eppendorf tube (2 mL) and evaporated to dryness under nitrogen in a thermoblock at 40°C. The dried residue was reconstituted into 200 *µ*L of methanol and was ready for injection onto the chromatographic column.

## 3. Results

In order to obtain the best extraction conditions for isolation of the five studied pesticides from oil samples, several experimental factors such as the volume of graphene suspension, amount of the salt (NaCl), type and volume of the desorbing solvent, desorption time with and without application of sonication energy, and desorption temperature were studied. The optimization process was conducted using the method of one independent variable. The extraction recovery (ER) for each pesticide was calculated from the following equation:

(1) $$\begin{array}{c} \text{ER}\left(\%\right)=\frac{P_{\text{pesticide in sample extract}}}{P_{\text{pesticide standard}}}\cdot f_{1}\cdot f_{2}\cdot 100, \end{array}$$

where *P*_pesticide in sample extract_ is the area of the peak corresponding to the pesticide in a sample extract, *P*_pesticide standard_ is the area of the peak corresponding to the pesticide in a standard solution, *f*_1_ is the ratio of theoretical volume of an acetonitrile supernatant above the frozen fat layer to volume of an acetonitrile supernatant taken from above the frozen fat layer, *f*_2_ is the ratio of theoretical volume of an acetonitrile supernatant after desorption to volume of an acetonitrile supernatant after desorption, taken for evaporation.

For standardizing the extraction procedure, the acetonitrile supernatant volumes were taken to be the same, that is, 3.5 mL of the supernatant from above the frozen fat layer and 4 mL of the supernatant after the desorption process. Thus, values of the coefficients *f*_1_ and *f*_2_ are equaled to 1.14 and 1.25, respectively. As the optimization criterion (OC), the maximum average recovery rate for the five pesticides was adopted:

(2) $$\begin{array}{c} \text{OC}=\frac{1}{5}\cdot \sum_{i=1}^{n=5}{\text{ER}}_{i}=\text{maximum}, \end{array}$$

where *i* is the pesticide number and *n*=5 is the number of the studied pesticides.

### 3.1. Effect of Graphene Suspension Volume

Graphene was used as an adsorbent for the five pesticides. This nanostructured material seems to be an excellent adsorbent due to its huge surface area, as well as the hexagonal arrays of carbon atoms that are suitable for strong interactions with organic molecules, especially those possessing aromatic rings including the pesticides. The very large delocalized *π*-electron system enables the formation of strong *π*-*π* stacking interactions with aromatic rings [23].

To reduce errors when adding graphene to samples and to make the operation easy, graphene was prepared in the form of aqueous suspension. The amount of graphene was optimized at the five volume levels of the graphene suspension: 3, 4, 5, 6, and 7 mL. The remaining conditions such as the mass of NaCl (2 g), type and volume of the desorbing solvent (4 mL), sonication, desorption time (10 min), and temperature (ambient) during the desorption process were left constant. From this optimization step, the extraction results are shown in Table 1.

The maximal average value of extraction recovery for the five compounds was obtained for 5 mL of aqueous graphene suspension, and this volume was used for further study.

### 3.2. Effect of Desorbing Solvent Type

Four different solvents such as acetonitrile, acetone, ethyl acetate, and a mixture of *n*-hexane and dichloromethane (2 : 1, v/v) were tested as the desorbing agents for the studied pesticides from graphene. The choice of the tested solvents was based on their polar/hydrophobic properties. The hydrophobicity of the solvents along with their elution strength in relation to the studied pesticides, adsorbed on graphene, increases in the following sequence: acetone, acetonitrile, ethyl acetate, and a mixture of *n*-hexane and dichloromethane (2  1, v/v). The remaining conditions of the volume of the graphene suspension (5 mL), amount of NaCl (2 g), volume of the desorbing solvent (4 mL), sonication, desorption time (10 min), and temperature (ambient) during desorption were left constant. Considering the ease of experimental operations (very good separation of the organic supernatant and the graphene phase), the best solvent appeared to be ethyl acetate. The maximal extraction recovery for all of the pesticides was achieved with acetonitrile. Therefore, acetonitrile was selected as the desorbing solvent for further optimization process. The effect of the type of the used solvent is shown in Table 2.

### 3.3. Effect of Desorbing Solvent Volume

The effect of the five different volumes of acetonitrile (3, 4, 5, 6, and 7 mL) as the desorbing solvent on the extraction results of the five pesticides was examined. The remaining conditions of the volume of the graphene suspension (5 mL), amount of NaCl (2 g), type of the desorbing solvent (acetonitrile), sonication, desorption time (10 min), and temperature (ambient) during desorption were left constant. The highest extraction recovery for all the analytes was obtained using 5 mL of acetonitrile, and this volume was used in further experiments. The effect of this parameter is shown in Table 3.

### 3.4. Effect of Sonication Energy and Desorption Time

For desorbing the five pesticides from graphene, 5 mL of acetonitrile was added to the graphene phase (with the adsorbed analytes) at the bottom of a tube and vortexed for 1 min and then subjected to sonication energy for 5 and 10 minutes. Additionally, in a separate experiment, the graphene phase was vortexed (1 min) without applying sonication energy. The rest of the experimental conditions (volume of graphene suspension  (5 mL) and ambient temperature during desorption) were kept constant. A desorption process of 10 min gave the greatest extraction recovery. The effects of these experiments on extraction results are given in Table 4.

### 3.5. Effect of the Salt Added

In all the above experiments, before introduction of the graphene suspension to an oil sample, 2 g of NaCl was added for salting out of the pesticides. In this optimization step, the extraction efficacy of the analytes was studied by decreasing the amount of salt added to 1 g or performing the sample preparation procedure without addition of NaCl. The rest of the experimental parameters (volume of graphene suspension  (5 mL), acetonitrile volume (5 mL), sonication (10 min), and ambient temperature during desorption) were kept constant. The results are given in Table 5.

### 3.6. Effect of Desorption Temperature

The final experimental factor studied was the temperature of the pesticide desorption process from graphene. In the first step, the graphene phase (with the adsorbed pesticides) was heated with 5 mL of acetonitrile in a thermoblock to a temperature of 40°C; in the second step, the content of the tube was subjected to sonication for 10 min, maintaining the temperature at 40°C. The rest of the experimental parameters (volume of graphene suspension (5 mL), acetonitrile volume (5 mL), and sonication (10 min)) were kept constant. The effect of increasing the desorption temperature and how it considerably improves extraction efficiency are shown in Table 6.

### 3.7. Evaluation of Linearity Range and Limit of Detection of the d-SPE (Graphene)-HPLC/UV Method

At the optimized extraction conditions, the linearity range and detection limit for each pesticide were determined.

**Figure 2:**
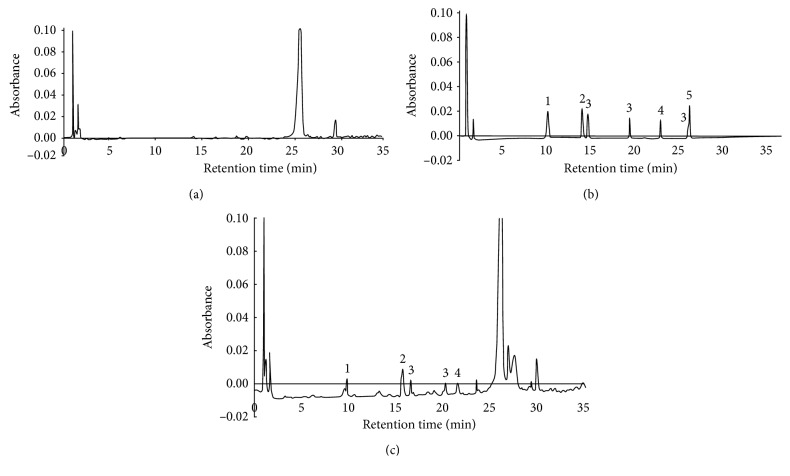
Chromatograms of the extract of the blank rape oil sample (a), five standard pesticides (1 *µ*g/1 ml of methanol) (b), and the extract of the rape oil sample, spiked with the five standard pesticides (c) (concentration (62.5 ng/g) equal to LOQ). 1: metazachlor; 2: tebuconazole; 3: *λ*-cyhalothrin; 4: chlorpyrifos; 5: deltamethrin.

Representative chromatograms are presented in Figure 2,

Calibration curves were designed on the basis of analyses of oil samples containing mixtures of the five pesticides at the six concentrations (62.5, 125, 250, 500, 1000, and 2000 ng·g^−1^) and the internal standard (chlorfenvinphos, 1 *µ*g·mL^−1^). A linearity range 62.5–2000 ng·g^−1^ was achieved for metazachlor, tebuconazole, *λ-*cyhalothrin, and chlorpyrifos with determination coefficients (*r* ^2^) 0.998, 0.991, 0.999, and 0.993, respectively. For deltamethrin, the linearity range was 500–2000 ng·g^−1^ with *r* ^2^ = 0.999. The detection limit was determined by estimation of the minimum concentration as equivalent to three times the background noise signal. The detection limit for metazachlor, tebuconazole, *λ-*cyhalothrin, and chlorpyrifos was 62.5 ng·g^−1^, and for deltamethrin, it was 500 ng·g^−1^.

## 4. Discussion

The potential adsorption properties of simple nonmodified graphene for the five pesticides (metazachlor, tebuconazole, *λ-*cyhalothrin, chlorpyrifos, and deltamethrin) analyzed from a rape oil matrix were explored. The fat content from the matrix was removed at two stages: (1) precipitation with acetonitrile and freezing at −32°C and (2) adsorption on the graphene surface (a smaller amount of oil left in the acetonitrile phase). The pesticides were adsorbed on graphene because of its high specific surface area, as well as through noncovalent interactions, especially the *π*-*π* stacking interaction with the aromatic rings of the studied compounds, and the hydrophobic effect.

The optimized d-SPE (graphene) extraction conditions for the five compounds present in rape oil samples were as follows: amount of graphene (15 mg), type of the desorbing solvent (acetonitrile), volume of the desorbing solvent (5 mL), desorption time using sonication energy (10 min), desorption temperature (40°C), and amount of salt (1 g NaCl). Under the optimized conditions, the following extraction recoveries for the examined pesticides were achieved: metazachlor (81%), tebuconazole (84%), *λ*-cyhalothrin (59%), chlorpyrifos (83%), and deltamethrin (98%). This is the first application of graphene-based materials in the analysis of pesticides in oil samples. There are relatively a small number of reports of carbon nanomaterials being used to extract pesticides from other less-complicated matrices including metazachlor from surface waters [24]; tebuconazole from environmental water [25], vegetable [20], and tea [22] samples; *λ*-cyhalothrin and deltamethrin from fruit [26] and vegetable [26, 27] samples; and chlorpyrifos from environmental water [14] and tea samples [22]. Depending on the kind of the pesticide or/and pesticide concentration and type of a matrix, the extraction recoveries of the pesticides of interest ranged from 79.0 to ca. 100%. The pesticides were separated and detected using the RP chromatographic system, which was adopted from literature [11] with slight modifications due to the tested pesticides. The detection limits of the five pesticides determined by the d-SPE (graphene)-HPLC/UV method were not quite satisfactory because some of them were above maximum residue levels (MRL; measured in rapeseeds [28]) for deltamethrin (MRL = 0.07 mg·kg^−1^), metazachlor (MRL = 0.06 mg·kg^−1^), and chlorpyrifos (MRL = 0.05 mg·kg^−1^). For *λ*-cyhalothrin (MRL = 0.2 mg·kg^−1^) and tebuconazole (MRL = 0.5 mg·kg^−1^), the developed method may be used as the so-called “cutoff” method allowing to separate “good” samples from “bad” oil samples. However, in our investigations, HPLC-UV, which is a relatively not highly sensitive technique, was primarily used as the detection tool for estimating the adsorption potential of graphene for the studied pesticides present in the oil matrix. In the abovementioned works, the detection limits achieved were considerably lower (single nanogram or less), but in the vast majority cases (with the exceptions of [25] and [27] where 300 mL of the aqueous sample was analyzed by HPLC-UV and a 10 g portion of the homogenized plant sample was assayed by GC-ECD, resp.), highly sensitive detection methods like mass spectroscopy (MS) or tandem mass (MS-MS) spectroscopy were used.

## 5. Conclusion

In most cases of pesticide analysis involving graphene as an adsorbent, magnetic solid-phase extraction (MSPE) was applied where magnetically modified graphene composites as adsorbents were used. Only few works reported the employment of nonmodified graphene alone or in combination with other sorbents (in the modified QuEChERS method) using dispersive solid-phase extraction. This is the first time when graphene in its nonmodified form, using d-SPE, was studied as the adsorbent for the pesticides from four different groups present in such analytically difficult matrices as edible oil. Graphene may be successfully exploited in the preparation of edible oil samples for determination of pesticides from various groups. However, some improvements should be introduced in such analyses. For example, a more sensitive analytical method (e.g., GC-MS or LC-MS) may be used. Additionally, a more suitable modified graphene form, for example, magnetically modified graphene, which will prevent the nanomaterial aggregation, increase its dispersion in solvents, and improve its adsorption properties, would better enable separation of graphene from the isolated analytes in the supernatant using an external magnet.

## Figures and Tables

**Table 1:** Effect of the graphene amount on extraction efficacy of the five pesticides from rape oil samples.

Pesticide	Extraction recovery (%), *n*=3				
	9 mg of graphene	12 mg of graphene	15 mg of graphene	18 mg of graphene	21 mg of graphene
Metazachlor	11 ± 3	64 ± 7	**68** **±** **5**	26 ± 5	14 ± 5
Tebuconazole	38 ± 8	72 ± 5	**77** **±** **5**	44 ± 7	29 ± 7
*λ*-Cyhalothrin	56 ± 7	52 ± 6	**60** **±** **5**	38 ± 11	17 ± 8
Chlorpyrifos	12 ± 7	63 ± 9	**66** **±** **7**	49 ± 7	23 ± 6
Deltamethrin	13 ± 5	63 ± 11	**68** **±** **9**	18 ± 4	14 ± 5
OC^1^	26.0 ± 20.3	63.0 ± 7.1	**67.0** **±** **6.2**	35.1 ± 12.6^2^	19.4 ± 6.5^2^

^1^Value of the optimization criterion; ^2^statistically significant from the highest result.

**Table 2:** Effect of the desorbing solvent type on extraction efficacy of the five pesticides from rape oil samples.

Pesticide	Extraction recovery (%), *n*=3			
	Acetonitrile	Acetone	Ethyl acetate	Dichloromethane/hexane (1 : 2,v/v)
Metazachlor	**68** **±** **6**	33 ± 5	61 ± 8	43 ± 6
Tebuconazole	**74** **±** **7**	22 ± 5	25 ± 5	34 ± 8
*λ*-Cyhalothrin	**60** **±** **6**	20 ± 4	50 ± 6	42 ± 6
Chlorpyrifos	**66** **±** **6**	42 ± 3	8 ± 7	63 ± 6
Deltamethrin	**66** **±** **5**	21 ± 3	39 ± 5	34 ± 5
OC^1^	**66.7** **±** **5.0**	27.7 ± 9.5^2^	36.9 ± 20.8^2^	43.5 ± 11.8^2^

^1^Value of the optimization criterion; ^2^statistically significant from the highest result.

**Table 3:** Effect of the desorbing solvent volume on extraction efficacy of the five pesticides from rape oil samples.

Pesticide	Extraction recovery (%), *n*=3				
	3 mL of acetonitrile	4 mL of acetonitrile	5 mL of acetonitrile	6 mL of acetonitrile	7 mL of acetonitrile
Metazachlor	18 ± 4	54 ± 6	**68** **±** **6**	29 ± 8	15 ± 3
Tebuconazole	38 ± 5	70 ± 5	**76** **±** **5**	48 ± 6	29 ± 5
*λ*-Cyhalothrin	34 ± 6	54 ± 7	**65** **±** **5**	32 ± 9	16 ± 5
Chlorpyrifos	22 ± 6	61 ± 10	**69** **±** **8**	51 ± 5	25 ± 7
Deltamethrin	16 ± 4	60 ± 5	**67** **±** **6**	21 ± 8	12 ± 5
OC^1^	25.9 ± 9.8^2^	59.8 ± 6.7	**69.2** **±** **4.2**	36.2 ± 12.9^2^	19.7 ± 7.1

^1^Value of the optimization criterion; ^2^statistically significant from the highest result.

**Table 4:** Effect of the sonication energy and desorption time on extraction efficacy of the five pesticides from rape oil samples.

Pesticide	Extraction recovery (%), *n*=3		
	10 minutes subjected to sonication	5 minutes subjected to sonication	Without sonication energy
Metazachlor	**68** **±** **8**	24 ± 4	37 ± 9
Tebuconazole	**74** **±** **7**	9 ± 4	43 ± 6
*λ*-Cyhalothrin	**60** **±** **7**	24 ± 7	32 ± 6
Chlorpyrifos	**66** **±** **6**	42 ± 8	63 ± 7
Deltamethrin	**66** **±** **7**	9 ± 5	10 ± 5
OC^1^	**67.0** **±** **4.8**	21.8 ± 3.7^2^	37.0 ± 19.1^2^

^1^Value of the optimization criterion; ^2^statistically significant from the highest result.

**Table 5:** Effect of the NaCl amount on extraction efficacy of the five pesticides from rape oil samples.

Pesticide	Extraction recovery (%), *n*=3		
	2 g NaCl	1 g NaCl	Without addition of NaCl
Metazachlor	68 ± 5	**69** **±** **6**	22 ± 6
Tebuconazole	74 ± 8	**73** **±** **8**	19 ± 4
*λ*-Cyhalothrin	60 ± 7	**63** **±** **4**	9 ± 5
Chlorpyrifos	66 ± 7	**70** **±** **9**	24 ± 5
Deltamethrin	66 ± 7	**68** **±** **7**	20 ± 5
OC^1^	66.8 ± 5.5	**68.5** **±** **4.2**	19.0 ± 6.5^2^

^1^Value of the optimization criterion; ^2^statistically significant from the highest result.

**Table 6:** Effect of the desorption temperature on extraction efficacy of the five pesticides from rape oil samples.

Pesticide	Extraction recovery (%), *n*=3	
	At ambient temperature	At increased temperature (40°C)
Metazachlor	69 ± 10	**81** **±** **5**
Tebuconazole	74 ± 8	**84** **±** **7**
*λ*-Cyhalothrin	63 ± 5	**59** **±** **5**
Chlorpyrifos	70 ± 6	**83** **±** **5**
Deltamethrin	68 ± 7	**98** **±** **8**
OC^1^	56.0 ± 3.8^2^	**80.9** **±** **13.0**

^1^Value of the optimization criterion; ^2^statistically significant from the highest result.
